# Visualization of intervertebral disc degeneration in a cadaveric human lumbar spine using microcomputed tomography

**DOI:** 10.1111/joa.13105

**Published:** 2019-10-31

**Authors:** Sascha Senck, Klemens Trieb, Johann Kastner, Stefan G. Hofstaetter, Herbert Lugmayr, Gunther Windisch

**Affiliations:** ^1^ University of Applied Sciences Upper Austria Wels Austria; ^2^ Orthopädische Abteilung Klinikum Wels‐Grieskirchen Wels Austria; ^3^ Institut für Radiologie I Klinikum Wels‐Grieskirchen Wels Austria; ^4^ Institut für makroskopische und klinische Anatomie Graz Medizinische Universität Graz Graz Austria

**Keywords:** annular fissure, endplate collapse, intervertebral disc degeneration, magnetic resonance imaging, microcomputed tomography

## Abstract

Gross features of disc degeneration (DD) that are associated with back pain include tears in the anulus fibrosus, structural changes of the endplates, and a collapse of the anulus. The aim of this study is the detailed visualization and microstructural characterization of DD using microcomputed tomography (μCT) and a dedicated image post‐processing pipeline. In detail, we investigate a cadaveric spine that shows both types of DD between L1 and L2 and between L2 and L3, respectively. The lumbar spine was obtained from a male donor aged 74 years. The complete specimen was scanned using μCT with an isometric voxel size of 93 μm. Subsequently, regions of interest (ROI) were prepared featuring each complete intervertebral disc including the adjacent endplates. ROIs were then additionally scanned with a voxel size of 35 μm and by means of magnetic resonance imaging. The collapsed endplate of the superior L2 showed explicit signs of an endplate‐driven degeneration, including bony endplate failures. In contrast, the intervertebral disc between L2 and L3 showed indications of an annulus‐driven DD including severe disc height loss and concentric tears. Using μCT we were able to visualize and quantify bone and cartilage features in DD. We showed that in both cases a suite of structural changes accompanies cartilage degeneration, including microstructural bony adaptions to counteract changes in the biomechanical loading regimen.

## Graphical abstract

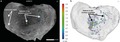

## Introduction

Degenerative changes of intervertebral discs, i.e. fibrocartilage pads that lie between the rigid vertebral bodies of the spine, are a major cause of back pain and frequent disability in the elderly (Adams & Dolan, 2012). Pathological changes of the intervertebral disc, commonly represented by fissures in the anulus fibrosus, degradation of the vertebral body endplates, and collapse of the anulus can lead to pronounced disc narrowing (Adams et al. 2014).

Basic causes of disc degeneration comprise genetic inheritance, age, inadequate metabolite transport, and loading regimens. Due to excessive mechanical loading, the intervertebral disc structure is affected, triggering a series of cell‐mediated responses, eventually causing further disruption (Adams & Roughley, 2006). A common symptom related to lumbar DD is low back pain. In patients showing chronic back pain, nerve fibers can penetrate the inner anulus fibrosus and nucleus pulposus (Freemont et al. 1997). The ingrowth of nociceptive nerves can be accompanied by blood vessels but the exact etiology is multi‐factorial and poorly understood (García‐Cosamalón et al. 2010).

Generally, degraded cartilaginous tissue progressively stiffens due to non‐enzymatic glycation (Verzijl & DeGroot, 2002; Smith & Fazzalari, 2009). The resulting fibrous and dehydrated nucleus is characterized by focal lamellar thickening and extensive lamellar disorganization (Berlemann et al. 1998). Variations in water content in the aging intervertebral disc lead to concentrations of focal compressive stresses that are regionally concentrated in the anulus (Adams et al. 1996). The heterogeneous distribution of areas that are exposed to varying degrees of compressive stresses is one factor influencing the propagation of ruptures (Galante, 1967).

Two types of disc degeneration have been proposed by Adams & Dolan (2012): endplate‐driven and annulus‐driven. Several features are characteristic for endplate‐driven disc degenerations, e.g. damaged endplates, circumferential tears between lamellae of the anulus, and an internal bulging or collapse of the anulus into the nucleus (Adams & Dolan, 2012). The visualization of an internal collapse of the anulus in living patients is difficult but Gunzburg et al. (1992) were able to detect endplate‐driven disc degenerations using magnetic resonance imaging (MRI) in cadaveric specimens. A marker for endplate‐driven defects is a reduction in disc space height. According to Adams & Dolan (2012), endplate‐driven disc degenerations are relatively rare in the lower lumbar spine. In the upper lumbar region, Schmorl's nodes are most common at the upper two lumbar levels and are specifically linked with severe disc degeneration at L1/2 and L2/3 (Mok et al. 2010).

In contrast, a pronounced disc height loss due to annulus‐driven disc degenerations is most frequently observed in the lower lumbar spine (Weiler et al. 2012). Degenerated lumbar intervertebral discs often show structural lesions in the posterior anulus fibrosus that can lead to radial fissures (Adams & Hutton, 1983). Radial fissures originate from within the nucleus and usually advance posteriorly or posterolaterally (Adams & Hutton, 1983). On MRI scans, radial fissures are visible as high intensity zones if they are filled with nucleus pulposus material (Finch, 2006). The formation of radial fissures can most frequently be observed in the lower lumbar spine, posterior to the nucleus, and its prevalence increases in the elderly population (Schwarzer et al. 1995).

Here we present the results of a microcomputed tomography (μCT) analysis of a cadaveric specimen that shows indications of an endplate‐driven disc degeneration between L1 and L2 and an annulus‐driven disc degeneration between L2 and L3. Several μCT studies showed the value of in‐depth three‐dimensional characterizations of endplate and vertebral disc morphology and pathologies (Rutges et al. 2011), but most of these studies focused on non‐human animals in controlled experiments (Gao et al. 2015). To our knowledge, this is the first detailed study that investigates pathological changes in the complete disc‐endplate system of a male donor using an industrial μCT system. Using optimized scanning parameters, the application of staining agents to enhance image contrast of soft tissue was not necessary. Therefore, we were able to visualize degenerative bone and cartilage features in a volume of interest in a near‐physiological condition. To compare our results with the clinical gold standard in spine imaging, we conducted an MRI scan of the cadaveric spine.

## Materials and methods

Here we show that using μCT we are able to visualize and quantify intervertebral disc degeneration, e.g. concentric (circumferential) tears and annular fissures, in a cadaveric lumbar spine. The lumbar spine was obtained from a cadaver aged 74 years housed at the biobank of the Medical University in Graz. The complete specimen was defrosted at room temperature and scanned using a microcomputed tomography scanner with an isometric voxel size (VS) of 93 μm with a RayScan 250E cone beam μCT device equipped with a Perkin Elmer flat panel detector (2048 × 2048 pixels with a pixel size 200 μm) and a Viscom 225 kV microfocus X‐ray tube. The X‐ray settings used were set to 190 kV and 480 μA with an integration time of 1000 ms. In total, 720 images were acquired using a 0.5‐mm copper filter to prevent beam‐hardening artifacts. Immediately after the scan, the complete piece was cut into five single pieces consisting of the intervertebral disc and the respective endplates. These pieces were vacuum‐sealed and separately scanned with a VS of 35 μm. The X‐ray settings were standardized to 110 kV and 300 μA with an integration time of 2500 ms. In total, 900 images were acquired per scan. No filter was applied to increase image contrast for soft tissue, i.e. the intervertebral disc.

Subsequently, all pieces were jointly scanned using a 1.5 Tesla MRI scanner with a Synergy Spine coil (Siemens MAGNETOM Avanto fit; Erlangen, Germany). In the sagittal planes, the T1 intensity images were constructed with a TE/TR of 9.4/600 ms, and the T2 intensity images were constructed with a TE/TR of 90/3300 ms. Slice thickness was set to 4 mm. Since the single parts were prepared for high‐resolution μCT scans prior to MRI scanning, the single parts are not in perfect physiological position.

The μCT images were thresholded using the advanced surface determination function in volume graphics 3.1 and morphological operations (Buie et al. 2007) to segment bone tissue from cartilage. Two three‐dimensional regions of interest (ROI) were defined: (1) the inferior endplate of L1, the superior endplate of L2 and the respective intervertebral disc between L1 and L2, and (2) the inferior endplate of L2, the superior endplate of L3 and the respective intervertebral disc between L2 and L3. The superior and inferior borders of the intervertebral discs were defined by the endplates. Since contrast between disc and surrounding non‐calcified tissue was too low to separate automatically cartilage from connective tissue, the peripheral regions of the respective intervertebral disc was segmented manually in vgstudiomax 3.1, resulting in a ROI for the whole intervertebral disc.

We used an image post‐processing pipeline including denoising, adjustment of image brightness and contrast, and smoothing. Since unprocessed μCT images of the intervertebral disc between L1 and L2 were characterized by image noise, we applied an Anisotropic Diffusion filter for denoising volume data (number of iterations: 6, diffusion stop threshold: 24285). This filter effectively preserves strong edges and enhances the contrast of edges. Subsequently, a median filter was applied using lowpass filters to reduce the contrast and soften the edges of objects in an image. It reduces contrast but also tends to defocus the image. The image stack was processed in three dimensions, considering a neighborhood of 18 voxels with at least one common edge. Finally, we applied a Brightness/Contrast filter that modifies the image brightness by adding an offset to the image values (value set to 1) while the contrast is modified by multiplying the difference from the voxel values to the average image intensity (value set to 2). All steps were carried out in avizo 2019.2. Image data of the intervertebral disc between L2 and L3 showed less noise and were only post‐processed using an Anisotropic Diffusion filter and by adjusting brightness in vgstudiomax 3.1 using the transfer function tool.

To visualize pronounced pathological changes in relation to the collapse of the vertebral endplate, we color‐coded changes in the elevation of the endplate relative to its peripheral boundary. Setting the endplate boundary as zero, elevations or depressions of the endplate are shown in purple and red, respectively. This visualization was performed using the ‘interp’ and ‘filled.contour’ functions based on the extracted coordinates of the STL file of the superior endplate of L2. Computations were carried out in R (R Development Team). Similarly, we visualized the variation in disc height using the ‘wall thickness’ function in geomagic qualify 10 (3D Systems).

Using the function ‘defect detection’ in vgstudiomax 3.1, voids, i.e. regions with a low gray value, were segmented and labeled according to their respective diameter in millimeters. Doing so, we were able to visualize defects in the degenerated nucleus pulposus in the L2/L3 disc. The segmentation of the protruded disc is based on Gauss 5 filtered and opacity mapped volume data (vgstudiomax 3.1).

Finally, we extracted morphometric parameters of the vertebral body of L2 and L3. To this end, we merged the respective parts that were obtained during sample preparation. Calculations are hence based on volume data with 35 μm VS, while the blank region of the separation was not considered. Calculated morphometric indices include bone volume fraction (BV/TV, bone volume/total volume), mean trabecular thickness (TbTh.mean), standard deviation of TbTh. (TbTh.SD), mean trabecular separation (TbSp.mean), standard deviation of TbSp. (TbSp.SD), and degree of anisotropy (DA). The computation of these indices was implemented in ctan (Version 1.16; Bruker) and is based on the work of Hildebrand & Ruegsegger (1997). Trabecular thickness was additionally visualized using the ‘wall thickness’ module available in vgstudiomax 3.1.

## Results

**Figure 1:**
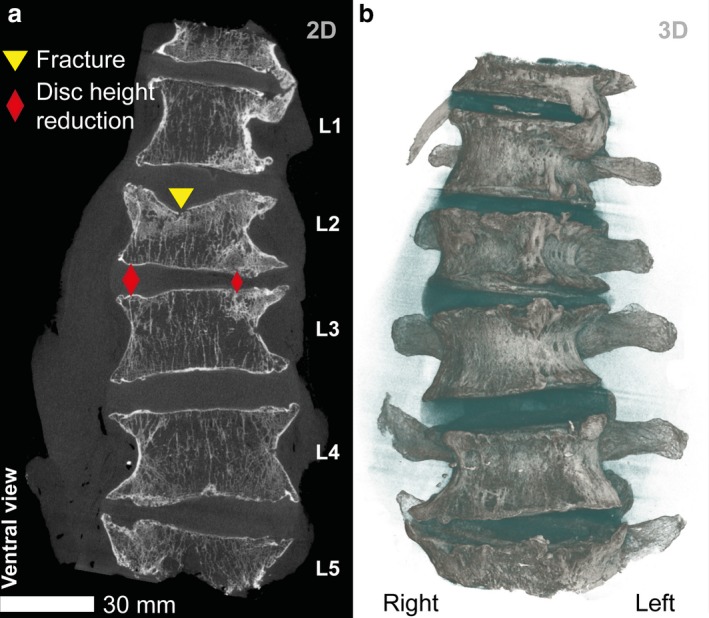
Coronal μCT slice (a) and 3D volume rendering (b) of the complete lumbar vertebral column (L1–L5; VS: 93 μm). The region of endplate collapse (triangle) in the superior L2 and the loss in disc space height (L2/L3; diamonds) are highlighted in (a). Intervertrebral discs in the threshold‐based volume rendering (b) are displayed in blue.

Fig. 1(a) shows the ventral view of a μCT slice and a 3D volume rendering (b) of the complete lumbar vertebral column (VS: 93 μm). At the level of T12 and L1, extensive osteophytes are present (claw osteophyte). Less pronounced osteophytes are present at L2 and L3. These are most probably the consequence of the scoliosis of the whole lumbar spine. Dominant features in the upper lumbar spine include the superior endplate of L2 that is characterized by a region of endplate collapse approximately 17 mm in diameter. Moreover, the intervertebral disc between L2 and L3 shows a severe loss in disc height. Those features indicate that the individual had suffered an age indeterminate vertebral compression fracture at L2 which appears to have healed.

**Figure 2:**
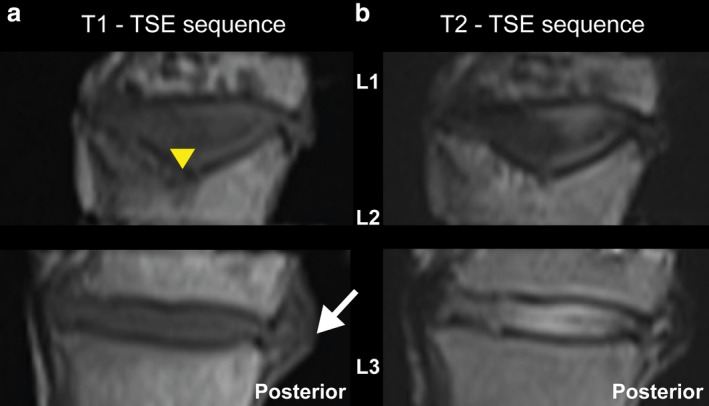
MRI scan (Turbo Spin Echo: TSE): (a) sagittal T1 and (b) T2. The intervertebral disc between L1 and L2 is expanded into the collapsed superior L2 (yellow triangle). A prolapse of the intervertebral disc between L2 and L3 can be detected in the T1‐weighted MRI scan (arrow). In the T2‐weighted MRI image, the intervertebral disc between L2 and L3 is characterized by an increased signal intensity.

A prolapse of the intervertebral disc between L2 and L3 can be detected in the T1‐weighted MRI scan (Fig. 2a). Furthermore, the height of the intervertebral disc is decreased. In the T2‐weighted MRI scan, the intervertebral disc between L2 and L3 is characterized by a hyperintense signal (Grade 2; Griffith et al. 2007). It can also clearly be seen that the intervertebral disc between L1 and L2 is expanded into the second lumbar vertebrae and that the superior endplate of L2 is eroded (Type 6 in the modified Pfirrmann grading system for lumbar DD, Griffith et al. 2007). In the sagittal view, both T1‐weighted and T2‐weighted images show a decreased signal intensity of subchondral bone marrow, indicating a replacement of bone with bony sclerosis (Type 3 Modic changes).

### Endplate‐driven intervertebral disc degeneration

**Figure 3:**
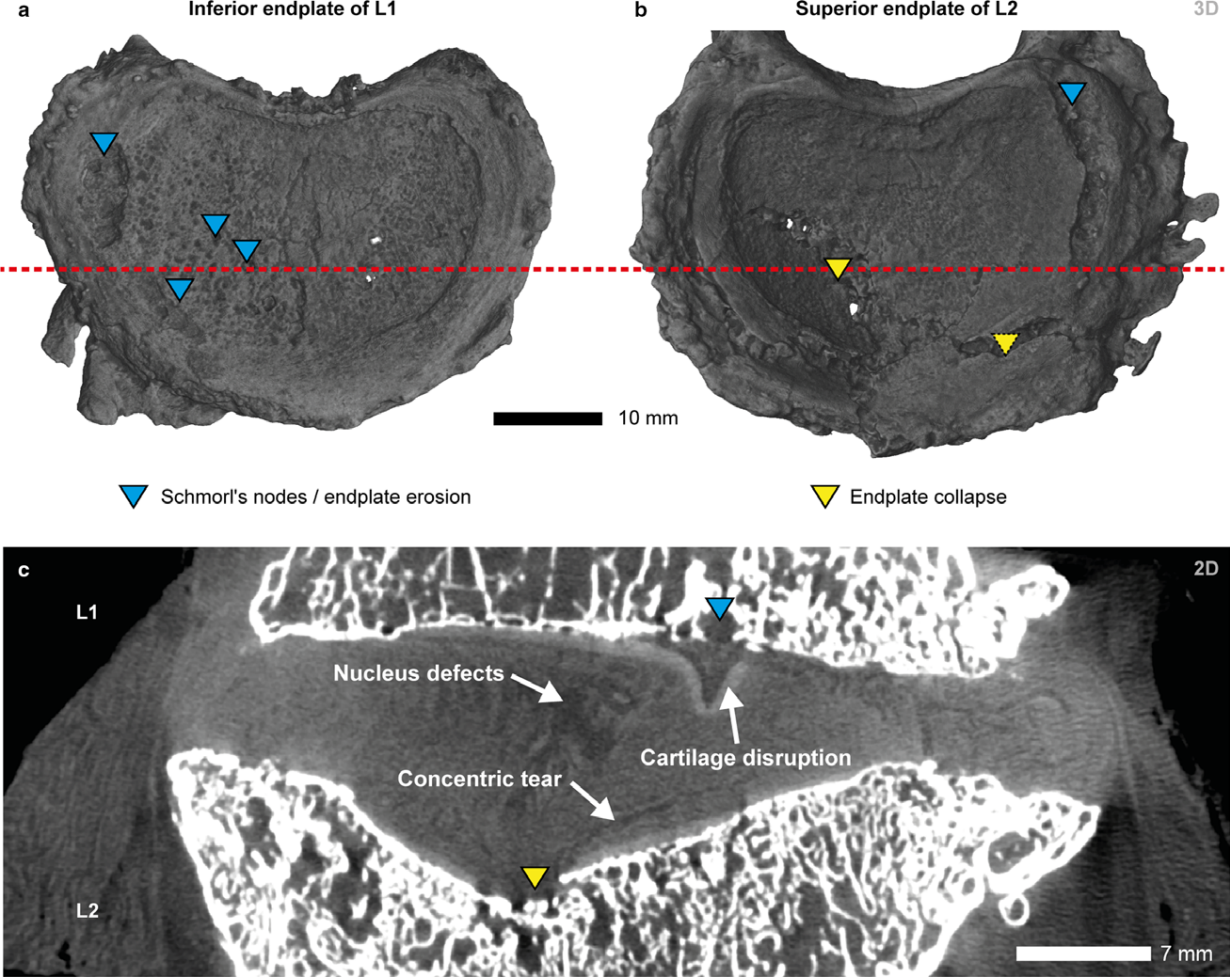
μCT volume renderings of the inferior endplate of L1 (a), superior endplate of L2 (b; right), and μCT coronal slice at the height of the red dashed line (c; VS: 35 μm). Both endplates are characterized by lesions (blue triangles) and two major collapses (yellow triangles). The intervertebral disc in (c) shows disrupted cartilage at the site of an endplate lesion, concentric annular tears, and defects in the nucleus.

Fig. 3(a) shows the inferior L1 endplate, characterized by extensive endplate defects, i.e. lateral Schmorl's node and endplate erosion. The most prominent feature of the intervertebral disc between L1 and L2 is the collapsed endplate of the superior L2, which shows explicit signs of an endplate‐driven degeneration (Fig. 3b). The bulging intervertebral disc occupies a large space in the superior aspect of L2 even though there are no signs of a disrupted disc. Nevertheless, concentric tears between the lamellae of the anulus and defects in the nucleus of the disc point to an extensive internal lamellar disorganization, particularly in the superior region (Fig. 3c). The superior central part of the intervertebral disc is detached from the endplate in the vicinity of an endplate lesion (see Fig. 3: arrow labeled as cartilage disruption).

**Figure 4:**
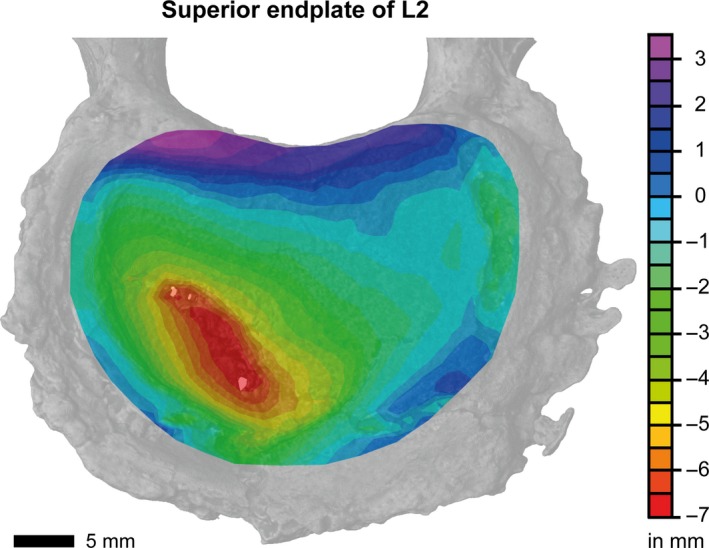
Vertical view of the three‐dimensional pseudo‐color image of the superior L2 endplate. The pseudo‐color bar indicates the depths of endplate fractures in relation to the unaffected endplate surface. The endplate fracture (red) is up to 7 mm below the endplate surface.

The pseudo‐colored image of the superior L2 endplate in Fig. 4 shows the extent of the endplate collapse displayed in Fig. 3. The collapsed endplate is consistent with an endplate‐driven DD. The maximum depth of the main depression is *ca*. 7 mm below the endplate surface (red area in Fig. 4).

**Figure 5:**
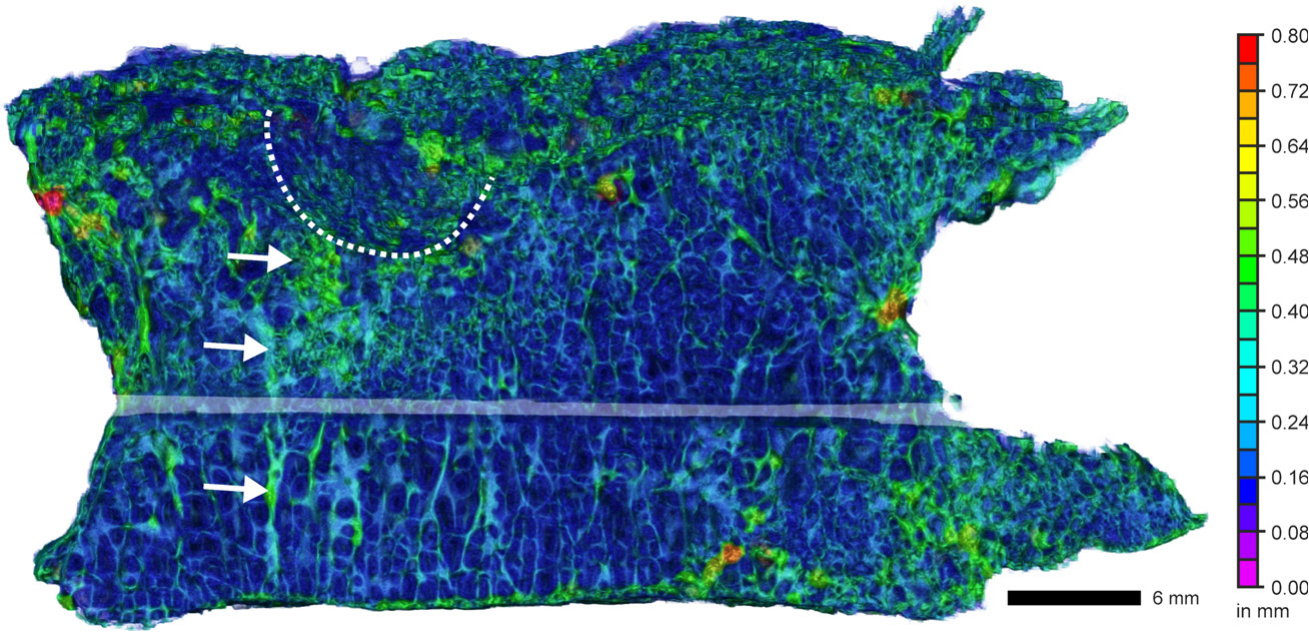
Trabecular thickness distribution in L2 (VS: 35 μm). The pseudo‐color bar indicates the wall thickness of trabeculae. Trabecular bone inferior to the endplate collapse (dashed line) is locally increased (arrows).

The distribution of trabecular thickness in L2 shows a local increase in Tb.Th. inferior to the endplate collapse (dashed line, Fig. 5). Trabecular microstructure in the immediate vicinity of the collapse seems to be less organized, showing thicker trabeculae with a decreased trabecular length. A similar pattern can be observed in the lateral osteophytes.

The values of the extracted morphometric parameters are presented in Table 1.

**Table 1:** Extracted morphometric parameters of L2 and L3. Each complete vertebral body consists of two merged parts (VS: 35 μm). The missing central cutting planes (see Figs 5 and 9) were not considered in the computation

Sample	BV/TV (in %)	TbTh.mean (in μm)	TbTh.SD (in μm)	TbSp.mean (in μm)	TbSp.SD (in μm)	DA
L2	25.56	279.78	153.56	810.61	413.75	0.32138
L3	17.69	272.66	167.80	1150.47	523.91	0.27260

### Annulus‐driven intervertebral disc degeneration

**Figure 6:**
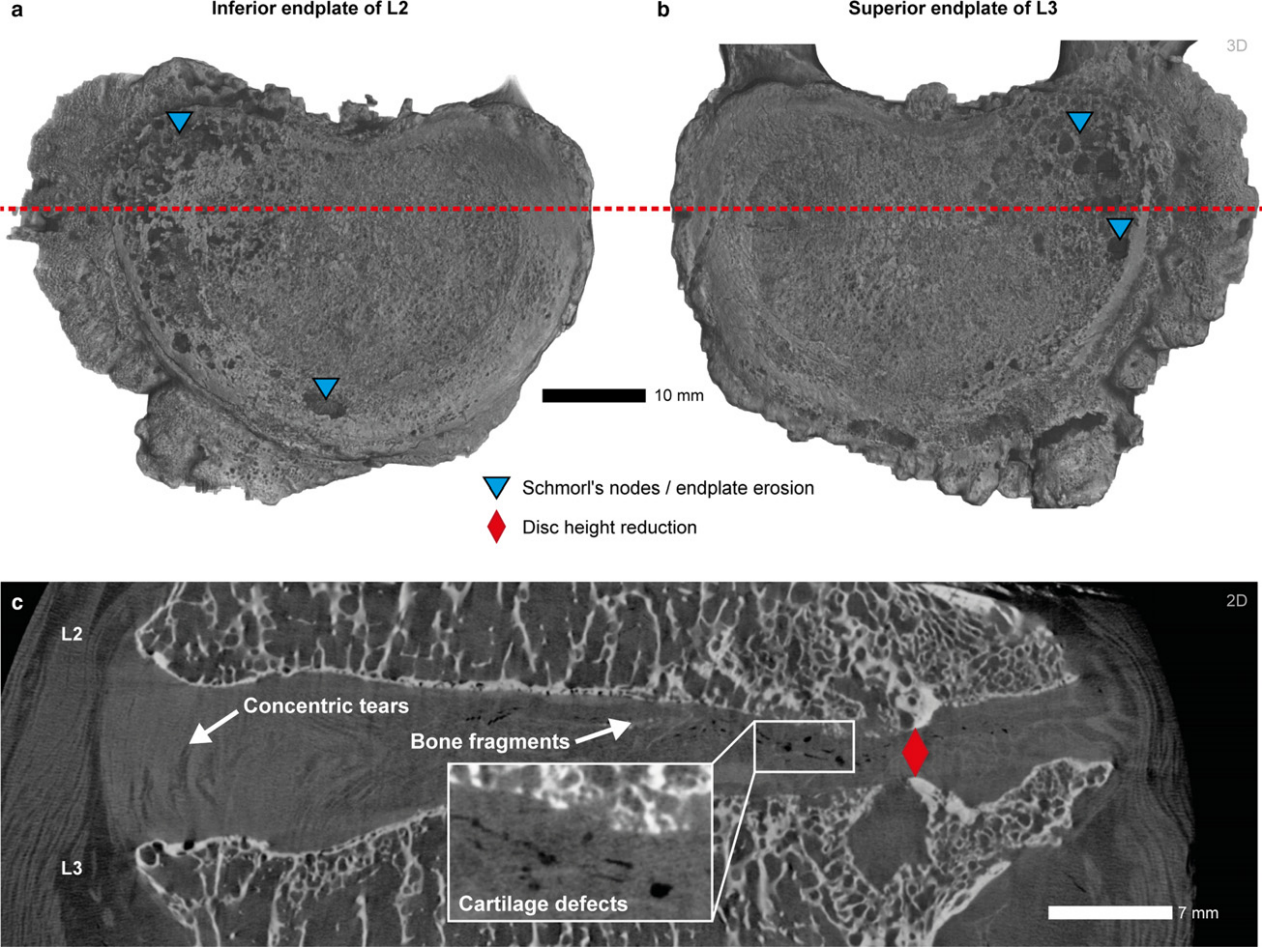
μCT volume renderings of the inferior endplate of L2 (a), superior endplate of L3 (b; right), and μCT coronal slice at the height of the red dashed line (c; VS: 35 μm). Both endplates are characterized by extensive osteophytes and lesions (blue triangles) at the side of decreased disc height (red diamond). The intervertebral disc in (c) shows a perforation of the nucleus pulposus at the side of protrusion, loose bone fragments at the L2 inferior endplate, and extensive concentric tears.

Both the inferior L2 and superior L3 endplate display extensive osteophytes and endplate defects at the side that is characterized by a decrease in disc height (Fig. 6a,b). The intervertebral disc between L2 and L3 shows indications of an annulus‐driven DD including severe disc height loss, concentric tears, and tube‐like cartilage defects (Fig. 6c).

**Figure 7:**
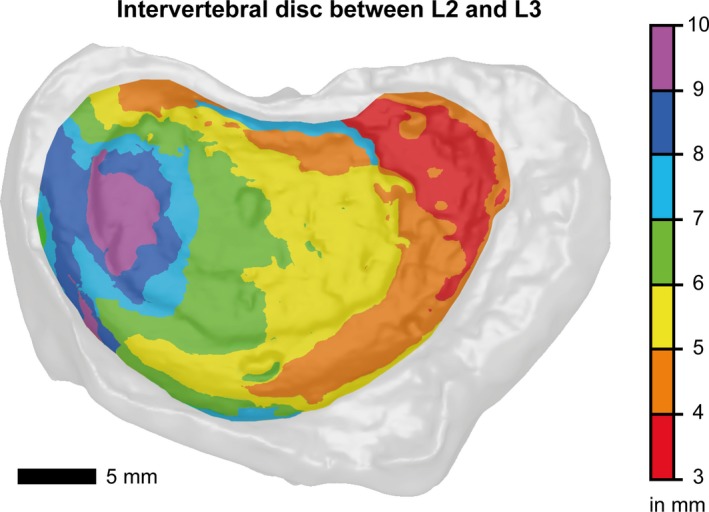
Thickness map (disc height distribution) of the intervertebral disc between L2 and L3. Regions of severe disc height reduction are shown in red.

The pseudo‐colored image of the intervertebral disc between L2 ‐ L2 in Fig. 7 shows the extent of the disc height reduction. The protruded disc displays a maximum height of ca. 10 mm and a minimum height of 3 mm at the side of disc height.

**Figure 8:**
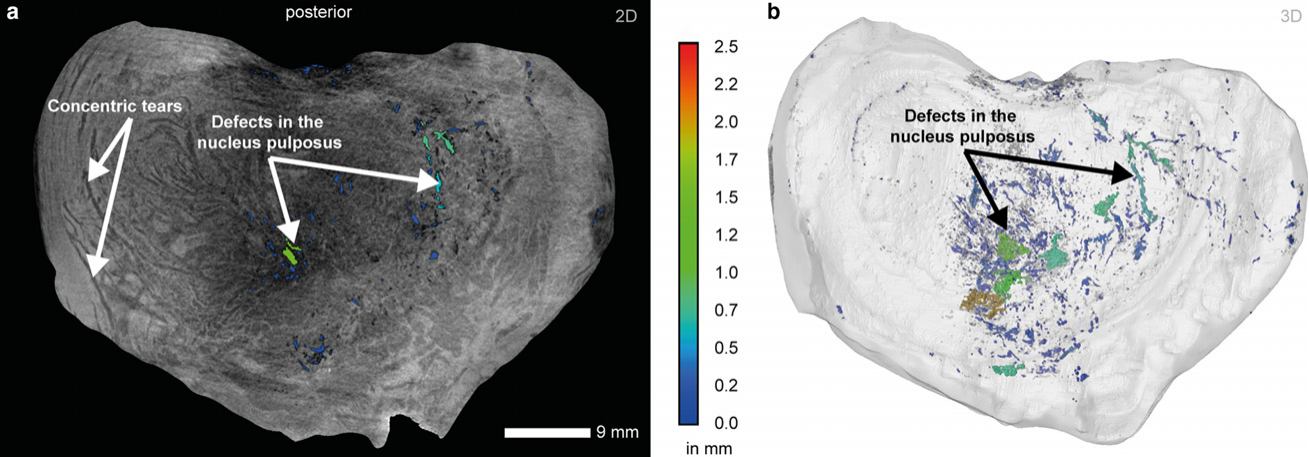
μCT transversal slice (a) and isosurface representation of the intervertebral disc between L2 and L3 showing labeled defects in the nucleus pulposus and the anulus fibrosus (VS: 35 μm). The color code corresponds to the maximum diameter of the respective labeled defect. (a) Concentric tears and defects in the nucleus pulposus. (b) Semi‐transparent surface rendering showing labeled defects. The largest defects occur in the central region of the nucleus pulposus.

Figure 8 displays the segmentation of loss of nucleus pulpous material and concentric tears in the intervertebral disc between L2 and L3. Concentric tears between the lamellae of the anulus can easily discerned due to the high contrast in the filtered and mapped volume data. On the side of disc height reduction several tube‐shaped defects with a length up to 10 mm merge into a single larger fissure that opens in the medial posterior region of the disc (protrusion). Those defects (see magnified view in Fig. 6c) are concentrated in the compressive loading area, whereas circumferential (concentric) tears can be found in the zone of increased disc height. The large defects in the central area of the nucleus pulposus may be due to the extrusion of the nucleus through the disrupted anulus.

**Figure 9:**
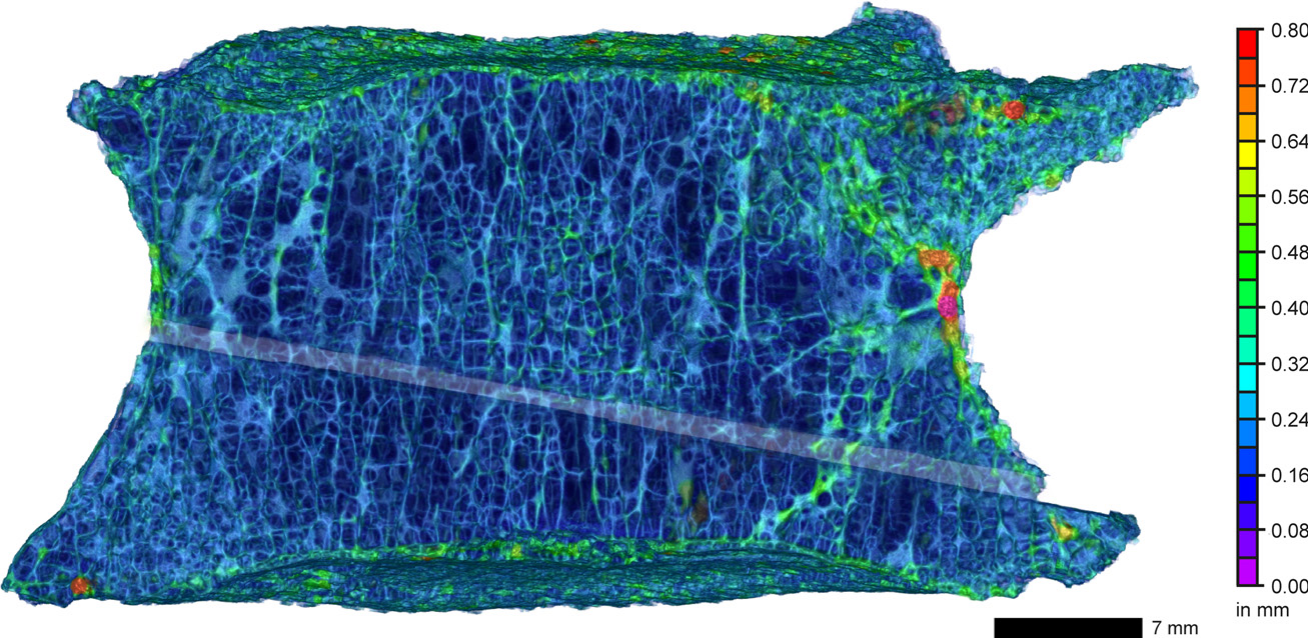
Trabecular thickness distribution in L3 (VS: 35 μm). The pseudo‐color bar indicates the wall thickness of trabeculae. Compared with L2, L3 shows a lower BV/TV and overall DA.

Trabecular thickness in L3 is homogeneously distributed in the vertebral body, showing a central region with more closely spaced trabeculae (see Fig. 9). Qualitatively, trabecular microstructure is less organized in the osteophytes, showing a decreased level of anisotropy in those regions compared with the central portion of the vertebral body. Extracted morphometric parameters are reported in Table 1. In contrast to L2, bone volume fraction (BV/TV) is significantly lower in L3 (17.69%) than L2 (25.56%). The lower level of BV/TV is accompanied by a high value of TbSp.mean and TbSp.SD, even though mean Tb.Th. is very similar in both vertebral bodies. Finally, both L2 and L3 show low levels of DA (Table 1).

## Discussion

Back pain is strongly correlated with radial fissures that advance in the direction of the peripheral disc (Smith & Fazzalari, 2009). The innervation of the anulus is normally confined to the peripheral 3 mm, although nerves can grow further in along radial fissures (Freemont et al. 1997; Stefanakis et al. 2012). Our results show that in both discs, concentric tears and defects in the nucleus lead to extensive internal lamellar disorganization and loss of disc height. Furthermore, both intervertebral discs display small Smorl's nodes (Figs 3 and 6. Based on MRI and μCT image data, it seems more likely that both discs underwent annular degeneration until an L2 compression fracture, rather than the compression fracture being the driver of the DD.

Due to optimized μCT scan parameters, we were able to visualize those defects without the need for staining agents. This allowed the three‐dimensional characterization of cartilaginous defects in reasonable detail, particularly in relation to defects in the nucleus pulposus and anulus fibrosus that are features of a disc protrusion between L2 and L3. We showed that DD is characterized by a complex of pathological changes including endplate damage, bone remodeling, radial fissures and concentric tears, and disc height reduction.

Using μCT we were able to visualize branching, tube‐shaped defects with a diameter of *ca*. 0.2 mm in a disrupted intervertebral disc that may be represented by the ingrowth of nociceptive nerves and accompanying blood vessels. A detailed investigation including sample preparation is desirable to discern the quality of those defects. However, the three‐dimensional structure of the disc during sample preparation may be extensively disturbed, complicating the detection of small‐scale features such as tears and fissures and their spatial distribution. Hence, the non‐destructive evaluation of defects in pathological intervertebral discs using μCT is a useful alternative analysis method that preserves the spatial morphology of sensitive samples.

We visualized in detail the vertical expansion of DD into the adjacent vertebral bodies (superior L2) as a consequence of a collapsed endplate. However, we did not quantify intervertebral disc height, since the extraction of the lumbar spine from the surrounding tissue and the extraction of the spinal cord altered the pattern of stresses, which will differ from the physiological condition. This also explains missing information on spinal stenosis. Hence, we are not able to speculate the extent to which two adjacent disc failures are structurally related. This attempt was further impeded by missing data concerning the donor's medical history, e.g. whether the individual had osteoporosis. Furthermore, μCT data were not calibrated and hence no information about apparent bone mineral density is available.

Since the quantification of vertebral bone morphometry leads to a deeper knowledge of the three‐dimensional microarchitecture, we extracted standard indices. BV/TV was 25.56% for L2 and 17.69% for L3, greater than the values for L2 of geriatric men (11.3 ± 4.0% reported by Lochmüller et al. (2008). Moreover, values for Tb.Th. and Tb.Sp. reported in Table 1 are lower than values reported by Lochmüller et al. (2008) (Tb.Th.: 155 ± 20 μm, Tb.Sp.: 93 ± 16 μm). In contrast to the majority of existing studies, we computed morphometric indices for the whole intervertebral body (including osteophytes) rather than extracting smaller ROIs in the center of the complete bone. It is therefore difficult to compare our results with those of other studies investigating vertebral microstructure. In this sample, DA values between 0.27 and 0.32 point to a rather low degree of anisotropy. Whereas highly organized trabecular bone is associated with higher anisotropic values, disordered bone deposition, e.g. of unorganized bone in osteophytes, is associated with decreased anisotropy.

The specimen investigated in our sample showed strong pathological changes in spine morphology including pronounced osteophytes, a scoliosis of the lumbar spine, and a collapse of the endplate at L2. Moreover, bone microstructure in the respective vertebral body showed strong differences in the spatial distribution of trabecular bone, e.g. inferior to the endplate collapse. Orthopedic surgeons confronted with such complicated cases face considerable challenges during surgical interventions, e.g. in relation to osteophytes and differences in the microstructural organization, i.e. material properties, of the bone. The information gathered in this study may hence be relevant in the development of precise anatomical models for the training of prospective spine surgeons using surgical simulators and artificial bone models (Fürst et al. 2017) and for the improvement of personalized implants, e.g. for additively manufactured spinal cages that are able to distribute peak loads more regionally.

## Conclusion

With advancing age, intervertebral discs frequently exhibit degenerative changes which are a major cause of back pain. Using μCT volume data of a cadaveric lumbar spine, we visualized internal defects of two disrupted intervertebral discs and accompanying bone lesions. We showed that disc degeneration is characterized by a complex suite of pathological changes including endplate damage, circumferential tears, and disc height reduction.

## Funding

This work was supported by the project ‘Com3d‐XCT’ (Interreg ATCZ38) funded by the European Regional Development Fund (EFRE) in the framework of the Interreg V program ‘Austria‐ Czech Republic’.

## Conflict of interest

All authors declare that they have no conflict of interest.

## Ethical approval

No formal consent is required for this type of study. The data that support the findings of this study are available from the corresponding author, Sascha Senck, upon reasonable request.

## References

[joa13105-bib-0001] Adams MA , Dolan P (2012) Intervertebral disc degeneration: evidence for two distinct phenotypes. J Anat 221, 497–506. 22881295 10.1111/j.1469-7580.2012.01551.x PMC3512277

[joa13105-bib-0002] Adams MA , Hutton WC (1983) The effect of fatigue on the lumbar intervertebral disc. J Bone Joint Surg Br 65, 199–203. 6826631 10.1302/0301-620X.65B2.6826631

[joa13105-bib-0003] Adams MA , Roughley PJ (2006) What is intervertebral disc degeneration, and what causes it? Spine (Phila Pa 1976) 31, 2151–2161. 16915105 10.1097/01.brs.0000231761.73859.2c

[joa13105-bib-0004] Adams MA , Nally DSMC , Dolan P (1996) ‘Stress’ distributions inside intervertebral discs. The effects of age and degeneration. J Bone Joint Surg Br 78, 965–972. 8951017 10.1302/0301-620x78b6.1287

[joa13105-bib-0005] Adams MA , Lama P , Zehra U , et al. (2014) Why do some intervertebral discs degenerate, when others (in the same spine) do not? Clin Anat 28, 195–204. 24753325 10.1002/ca.22404

[joa13105-bib-0006] Berlemann U , Gries NC , Moore RJ (1998) The relationship between height, shape and histological changes in early degeneration of the lower lumbar discs. Eur Spine J 7, 212–217. 9684954 10.1007/s005860050058 PMC3611258

[joa13105-bib-0007] Buie HR , Campbell GM , Klinck RJ , et al. (2007) Automatic segmentation of cortical and trabecular compartments based on a dual threshold technique for *in vivo* micro‐CT bone analysis. Bone 41, 505–515. 17693147 10.1016/j.bone.2007.07.007

[joa13105-bib-0008] Finch P (2006) Technology Insight: imaging of low back pain. Nat Clin Pract Rheumatol 2, 554–561. 17016481 10.1038/ncprheum0293

[joa13105-bib-0009] Freemont AJ , Peacock TE , Goupille P , et al. (1997) Nerve ingrowth into diseased intervertebral disc in chronic back pain. Lancet 350, 178–181. 9250186 10.1016/s0140-6736(97)02135-1

[joa13105-bib-0010] Fürst D , Senck S , Hollensteiner M , et al. (2017) Characterization of synthetic foam structures used to manufacture artificial vertebral trabecular bone. Mater Sci Eng, C 76, 1103–1111. 10.1016/j.msec.2017.03.158 28482474

[joa13105-bib-0011] Galante JO (1967) Tensile properties of the human lumbar annulus fibrosus. Acta Orthop Scand Suppl 100, 1–91. 10.3109/ort.1967.38.suppl-100.01 6040333

[joa13105-bib-0012] Gao C , Chen BP , Sullivan MB , et al. (2015) Micro CT analysis of spine architecture in a mouse model of scoliosis. Front Endocrinol 6, 1–9. 10.3389/fendo.2015.00038 PMC4365746 25852647

[joa13105-bib-0013] García‐Cosamalón J , del Valle ME , Calavia MG , et al. (2010) Intervertebral disc, sensory nerves and neurotrophins: who is who in discogenic pain? J Anat 217, 1–15. 20456524 10.1111/j.1469-7580.2010.01227.x PMC2913007

[joa13105-bib-0014] Griffith JF , Wang YXJ , Antonio GE , et al. (2007) Modified Pfirrmann grading system for lumbar intervertebral disc degeneration. Spine (Phila Pa 1976) 32, E708–E712. 18007231 10.1097/BRS.0b013e31815a59a0

[joa13105-bib-0015] Gunzburg R , Parkinson R , Moore R , et al. (1992) A cadaveric study comparing discography, magnetic resonance imaging, histology, and mechanical behavior of the human lumbar disc. Spine (Phila Pa 1976) 17, 417–426. 1579876 10.1097/00007632-199204000-00007

[joa13105-bib-0016] Hildebrand T , Ruegsegger P (1997) A new method for the model‐independent assessment of thickness in three‐dimensional images. J Microsc 185, 67–75.

[joa13105-bib-0017] Lochmüller EM , Pöschl K , Würstlin L , et al. (2008) Does thoracic or lumbar spine bone architecture predict vertebral failure strength more accurately than density? Osteoporos Int 19, 537–545. 17912574 10.1007/s00198-007-0478-x

[joa13105-bib-0018] Mok FPS , Samartzis D , Karppinen J , et al. (2010) ISSLS prize winner: prevalence, determinants, and association of Schmorl nodes of the lumbar spine with disc degeneration: a population‐based study of 2449 individuals. Spine (Phila Pa 1976) 35, 1944–1952. 20838277 10.1097/BRS.0b013e3181d534f3

[joa13105-bib-0019] Rutges JPHJ , van der Jagt OP , Oner FC , et al. (2011) Micro‐CT quantification of subchondral endplate changes in intervertebral disc degeneration. Osteoarthritis Cartilage 19, 89–95. 20950699 10.1016/j.joca.2010.09.010

[joa13105-bib-0020] Schwarzer AC , Aprill CN , Derby R , et al. (1995) The prevalence and clinical features of internal disc disruption in patients with chronic low back pain. Spine (Phila Pa 1976) 20, 1878–1883. 8560335 10.1097/00007632-199509000-00007

[joa13105-bib-0021] Smith LJ , Fazzalari NL (2009) The elastic fibre network of the human lumbar anulus fibrosus: architecture, mechanical function and potential role in the progression of intervertebral disc degeneration. Eur Spine J 18, 439–448. 19263091 10.1007/s00586-009-0918-8 PMC2899476

[joa13105-bib-0022] Stefanakis M , Al‐Abbasi M , Harding I , et al. (2012) Annulus fissures are mechanically and chemically conducive to the ingrowth of nerves and blood vessels. Spine (Phila Pa 1976) 37, 1883–1891. 22706090 10.1097/BRS.0b013e318263ba59

[joa13105-bib-0023] Verzijl N , DeGroot J (2002) Crosslinking by advanced glycation end products increases the stiffness of the collagen network in human articular cartilage. Arthritis Rheum 46, 114–123. 11822407 10.1002/1529-0131(200201)46:1<114::AID-ART10025>3.0.CO;2-P

[joa13105-bib-0024] Weiler C , Schietzsch M , Kirchner T , et al. (2012) Age‐related changes in human cervical, thoracal and lumbar intervertebral disc exhibit a strong intra‐individual correlation. Eur Spine J 21, 810–818. 10.1007/s00586-011-1922-3 PMC3535216 21837413

